# Meaning of Education and Wellbeing: Understanding and Preventing the Risk of Loss of Meaning in Students

**DOI:** 10.3389/fpsyg.2022.796107

**Published:** 2022-02-28

**Authors:** Nadia Baatouche, Paul de Maricourt, Jean-Luc Bernaud

**Affiliations:** ^1^Laboratoire du Centre de Recherche sur le Travail et le Développement (CRTD), CNAM, EA4132, Paris, France; ^2^Independent Consultant, Fontenay-sous-Bois, France

**Keywords:** meaning of education, meaning, wellbeing, IPA (interpretive phenomenological analysis), reflexive approach, students

## Abstract

The phenomenon of malaise is on the rise at universities, reflecting a deteriorating psychological state that is a combination of anxiety and stress factors. This psychological and emotional upheaval within students is indicative of a fundamental existential issue. In fact, hidden behind the choice of an educational program is the significance given by the student to their life goals. It is this dimension of attributing meaning to one’s education and, more broadly, to one’s life (the existential dimension) that we have sought to explore. We hypothesized that a stable investment in one’s life goals and a sense of psychological wellbeing during one’s studies could be fostered by reflective work done alongside the educational process. Our research took the form of a mixed methodological approach to the attribution of meaning to education, including an interpretive phenomenological analysis (IPA), and the experimentation of support for the meaning of education. Four dimensions of meaning were found to be observable in varying degrees in all students, each playing a specific role. Moreover, this research has confirmed that the meaning of studies is not to be understood solely in terms of education, but is part of a singular life story. Reflective work, developing meaning, facilitated by others (advisor, teacher, etc.) can help preserve/restore the feeling of wellbeing. It should be noted that, as the work presented in this article predates the pandemic, we will not address the amplifying effects of this health crisis on existential issues, which some recent studies are beginning to highlight.

## Introduction

Evolving in a context of multiple crises and uncertainties (Coutinho et al., 2008; Arnault, 2021), and called upon to adapt quickly to the university environment (Boujut et al., 2009) regardless of their degree of autonomy, self-esteem, and emotional stability, students react mainly (and sometimes sequentially) in two ways: by fully engaging in the acquisition of knowledge and skills deemed useful for their career life, at the cost of excelling in their work (intensive investment), or by developing negative affects — boredom, stress, fear of failure, questioning of their own choices, etc., - which can lead to disengagement or even dropping out (Falissard, 2019). How can we maintain the student’s commitment or allow them to reinvest in a course that has become a source of psychological wellbeing? Considering that future plans - which have become non-linear - are now linked to existential questions and the meaning of work (Yalom, 2008; Bernaud, 2016, 2021), it seems essential to us to help the student adopt a reflective stance toward their experiences.

Thinking and thinking about oneself in a learning situation would contribute to a better understanding of one’s existence, to the development of new behaviors, less focused on the goal of obtaining a degree to the detriment of one’s psychological wellbeing, and closer to oneself, one’s needs, and one’s priorities in life (Henderson-King and Mitchell, 2011). What kind of support could help to ensure a serene (re)investment in university education? This is the question we tried to answer in a recent Ph.D. research project based on a mixed empirical approach, taking advantage of an interpretative phenomenological approach to build a device to support meaning (Baatouche, 2020).

### From School to University

Whether enrolled in initial or continuing education, the student is always a former high school student. For them, university studies will often appear to be a continuum or a renewed thread in relation to their schooling. They will nevertheless mark a break, requiring a reworking of the meaning attributed to learning.

On entering university, learning and the particular place in which it takes place still bear the imprint of the meaning previously attributed to school. This meaning of school has itself been drawn from inherited cultural capital (Bourdieu and Passeron, 1971), which is made up of parental educational practices, beliefs and values of the environment, etc.

Inherited cultural capital constitutes a mental, symbolic and cognitive tool that allows us to interpret the world in which we evolve from childhood (Baatouche, 2020). Taking a step back from our primary culture can be done at school and at university through a reflective and hermeneutic pedagogy, allowing for a distancing, a step back, a reworking of meaning (Dumont, 1968). Thus, Zakhartchouk (1999) sees the teacher as a potential “cultural facilitator,” guiding the learner in a in a reflexive approach allowing the elaboration of a new meaning.

Furthermore, according to Paivandi (2018), the young learners entering college must transition from being a high school student to being a college student. During this transition, they are called upon to change their relationship to the object of learning, their relationship to knowledge, and their way of learning. College constitutes not only a new way of conceiving knowledge, by requiring independence and intellectual engagement, but also a space for questioning the meaning of one’s education. The student must therefore undergo a real intellectual transformation.

While as a high school student, they were learning to comply with parental orders (utilitarian function) or for the pleasure of acquiring knowledge (epistemic function). Now, as a university student, they must choose a course of study based on their personal and professional life goals.

The direction, which was previously undefined or imaginary, must become clearer in order to sustain a long-term motivated effort. The student also becomes aware of a timeframe (quarter and academic year) in which this mobilization takes place so as to empower them to take charge of their lives.

During this period of study, the meaning attributed to the learning process is transformed through interaction with the social (outside the university) and educational environment (Bloomer and Hodkinson, 2000). Learning then combines as many meanings as the number of situations encountered.

As for older students, returning to their studies, armed with experience of professional life, they are experiencing another major form of transition. This transition, often linked to their professional development, leads to a redefinition of their motivations for studying and an upheaval in the way they invest time, learning and social relations.

In the end, always marked by a cultural heritage and a life history, the meaning comes from the links that the learner weaves between their education, the environment in which it takes place, its content, past experiences (of learning) and their future plans. Major bad experiences can impregnate the meaning attributed to education, leading to malaise. The establishment of an internal dialogue between the individual as a person and the individual as a learner will make it possible to sort out their thoughts, in order to clarify what led them to choosing this course of study, in order to persevere by having attributed a new meaning to their educational plans, or to change the course of study with full knowledge of the facts.

### Expressions of Malaise at Universities

Students live and search for their bearings in a fluid society (Bauman, 2013) marked by a continuum of transformations and experiencing a succession of crises (economic, philosophical, spiritual, and health). Finding meaning is all the more vital to them. According to Falissard (2019), the competitive world places students in a climate of permanent tension, resulting in either an appetite for challenge or unhappiness: stress, educational phobia, social and family isolation. Verger et al. (2010) note that approximately forty to fifty-six percent of students report a state of anxiety.

DeWitz et al. (2009) focus on students without a life plan. Being immersed in the academic environment leads them to question the meaning of their existence, developing a feeling of malaise. Therefore, students experience the university experience only through this feeling of too little existence. They focus on the passing of time and their belief in their abilities diminishes, sliding from boredom to more or less accentuated forms of depression.

The forms of unhappiness at universities are sometimes spectacular. Thus, the hikikomori phenomenon happens, voluntary home confinement which is characterized by social withdrawal, lasting from a few weeks to several years (housebound syndrome), becoming an exclusively virtual communication with the surrounding environment. This is a prevalent phenomenon in Japan where it affects 46% of students and is now also found in France (Fansten and Figueiredo, 2015). We are not referring to the confinement measures imposed, since the completion of this study, during the health crisis (also a source of isolation and discomfort for many students).

The intensity of engagement in the university environment can lead some workaholic students (Chamberlin and Zhang, 2009) to no longer meet their psychological needs. Other areas of life are neglected (Bovornusvakool et al., 2012). This overcommitment clearly detracts from student wellbeing. Libert et al. (2019) found a correlation between overcommitment (workaholism) and academic burnout.

These various phenomena, which reflect a deterioration in the relationship with learning at the university, are often the beginnings of a complete drop-out of a student who is suffering. They can be studied in terms of meaning (Baatouche, 2021; Baatouche et al., 2021).

### Meaning, a Key Determinant of Psychological Wellbeing

For Kasser and Ryan (1996), intrinsic motivation aimed at inner satisfaction (as opposed to extrinsic motivation, which is focused on materialistic goals and aims for reward) is a key factor in meaning attributed to academic training. According to the flow theory (Csikszentmihalyi and Csikzentmihaly, 1990), individuals who carry out a daily activity that is closely linked to their intrinsic motivations experience a high degree of meaning and wellbeing, enabling them to develop confidence and autonomy.

Henderson-King and Mitchell (2011) join this vision, while taking an existential approach: motivations (intrinsic or extrinsic) are linked to life experiences to which one has attributed a meaning that a reflexive approach can reveal and change.

Life events related to university learning play a role in building and finding meaning (King and Hicks, 2009). According to Gómez González et al. (2013), the meaning of education is not reduced to a search for meanings, but stems from an exploration of oneself, one’s life experiences, and one’s relationship with others and the world.

By facing new situations and environments, students are able to revise and refine the meaning they attribute to their education. However, this process can be perilous when support from a professional (teacher, advisor, etc.) is not provided. To encourage this metacognitive process which allows one to go beyond one’s first interpretations impregnated with affects, the use of narrative and more specifically the use of the explicitation interview is recommended (Vermersch, 2012).

The explanatory interview leads the student to describe his life experience (in particular his presence in education), to observe his ways of acting and to put them in connection with his judgments, his thoughts, the influences the environment in which it operates (Balas-Chanel, 2002). This reflective posture leads the student to a reflective awareness, leading him to go beyond his implicit, to give a more objective meaning to his actions and experiences. Analyzing your experience as a spectator is not a spontaneous act. Nonetheless, learning to learn from one’s life, that is to say to “thoughtful consciousness,” makes it possible to give meaning to what seems to be lacking (Vermersch, 2019).

Each student can assign meaning to their education (DeWitz et al., 2009). To do so, they must have the courage to face their freedom to act, choose to reinvest the meaning of their attendance at a course in an authentic way and persevere (excelling) or revise their plans (reinvesting themselves elsewhere), and in both cases take responsibility for it. This choice of authenticity, which comes under the heading of a work of better self-knowledge with a view to fully engage one’s existence (Jaspers, 1989), proves to be a factor of wellbeing (Forest et al., 2012; Bernaud et al., 2019).

The meaning of education experienced through a reflective process in fact generates a feeling of wellbeing (Guimard et al., 2015). It should be noted that there are two forms of wellbeing: subjective wellbeing and psychological wellbeing. Subjective wellbeing is characterized, according to Diener (1984), as the sum of an affective component, a cognitive component and a feeling of life satisfaction. Psychological wellbeing, which is oriented toward an existential perspective and takes into account the way in which the individual interacts with the world, is made up, according to Ryff (1989), of six components: a perception of autonomy, self-acceptance, a perception of mastery over the environment, a degree of personal accomplishment, positive relationships with others and life goals.

Futhermore, the psychometric instrument Questionnaire for Eudemonic Wellbeing “QEWB” (Waterman and Waterman, 1972) offers an assessment of meaning as an indicator of the wellbeing of students. Tested on a sample of *N* = 7,334 students, it consists of four items related to the notion of wellbeing.

## Objectives and Hypotheses

September 2018 through January 2020, we conducted a study whose main objective was to analyze and understand, from an existential perspective, the psychological resources that students can activate in order to attribute meaning to their training. Our wish was to propose to professionals an approach to vocational guidance centered on meaning and reflexivity.

We postulate two hypotheses:

*Hypothesis* 1: Seeking the meaning of an education through a reflective guidance would increase the student’s sense of psychological wellbeing by promoting more authentic choices.

*Hypothesis* 2: A reflective approach to one’s life experiences would enable the student to clarify their choices and attribute a high level of meaning to their education.

To verify our hypotheses, we chose to conduct two-phase research: an analysis phase and an experimental phase (Baatouche, 2021). The first phase consisted of exploratory research conducted on a sample of five students, based on an interpretative phenomenological analysis (IPA). The objective was to identify, through an analysis of life experiences and in particular learning experiences, the meanings attributed to the education. Then, questioning the impact of an accompaniment to the meaning on the serene (re)investment of the university formation, we elaborated and tested a device of reflexive accompaniment, by constituting two independent samples of students, allowing a comparison (experimental sample *N* = 32 and control sample *N* = 11). Then, examining the mode of guidance in the sense that could enable a serene (re)investment in university education, we developed a reflective device with two independent samples, making a comparison possible (experimental sample *N* = 32 and control sample *N* = 11). The objective of this second phase was to measure the effects of reflective guidance on the development of meaning in life and education, vocational choices and wellbeing.

## Methodology

### Choosing a Mixed Approach

In order to shed light on the meanings that a student attributes to their attendance in education, we chose a qualitative method: the interpretative phenomenological analysis (IPA). This methodology which uses autobiography, promotes a process of objectification by gradually taking a distance. This movement allows the student to shed old meanings (linked to beliefs and presuppositions) and the affects that accompany them. The process thus makes it possible to approach the student “authentic individuality” (Husserl, 2004).

Originating in the therapeutic field and recently used in guidance research, IPA is an inductive method for understanding complex psychological phenomena (Smith et al., 2009; Baatouche, 2020; Bernaud, 2021). IPA, which uses the semi-structured, in-depth interview as a technique, requires the researcher to shed presuppositions and position the participant as co-researcher, having his own knowledge of himself. In this, this innovative approach differs from other meaning exploration methodologies, such as the Family Semantic Grid (Ugazio et al., 2018). Indeed, during the IPA, meaning themes are not pre-existing to the interviews; they are determined by the very content of the interviews.

In the end this approach has proven to be very appropriate in order to closely examine the major experiences that generate wellbeing or malaise; to better understand the meaning that the student attributes to his presence in education. To encourage this metacognitive process, the use of narrative and more specifically the use of the explanatory interview is recommended and to their existence, and to enable them to transform this meaning.

In addition to the individual IPA interviews, we then set up quantitative research based on the results of experimenting with an innovative method of support for the meaning of education. This method, entitled Meaning of Life - Meaning of Education (SVSF), was previously developed based on two methods of guidance, Meaning and Life Goals (Gómez González et al., 2013) and Meaning of Life, Meaning of Work (Bernaud et al., 2015).

Our Meaning of Life, Meaning of Education (SVSF) program, consisting of five group sessions and three individual interviews (approximately 22 h total), is an innovative approach to supporting meaning and plans through its reflective and pedagogical methodology [a hermeneutic approach, based on exchanges between peers and with the facilitators and on a personal, confidential logbook designed to record one’s thoughts (Baatouche et al., 2021)], focusing on existential soul-searching and on the meaning of one’s plans.

Thus, SVSF constitutes a reflective process that engages the student in the development of new knowledge about the self; its relationship to the self, to others, to time, to socio-emotional judgments (Sylvestre, 2013). This knowledge can guide current or future vocational choices (Boutin and Lamarre, 2008). The student distances himself from certain beliefs, for example that of being driven by determinism, and opens up to new choices, projects that take into account his way of being, his life history and values, and his limits (Allouche, 2012).

Our quantitative study made it possible to measure the presence of the effects of SVSF guidance on two dimensions: the dimension of the meaning of life and the dimension of the meaning of education.

### Participants

Two cohorts of students of all ages who are questioning the meaning of their education but who do not have a severe pathology (proven depression, addictions, etc.) made up this research between 2018 and 2020. More specifically, for this research, we have chosen to include in our cohorts students in initial and continuing education, these two college audiences who, during their educations, are sometimes mixed together and sometimes benefit from distinct classes.

The first cohort (IPA approach) consisted of five students who perceived their education as a foundational experience, their number corresponding to the recommendations of Smith and Osborn (2004) and Clarke (2010). These students, all of whom were female, were enrolled in a Master’s degree in humanities (*N* = 4) and a doctorate in automotive engineering (*N* = 1).

The second cohort (SVSF device) was composed of 43 students questioning the meaning of education, constituting the experimental sample (*N* = 32) and the control sample (*N* = 11). All the participants had similar socio-demographic characteristics. Thus, in terms of gender distribution, the majority of participants were women (69% for the experimental group and 73% for the control group). On the other hand, the average ages of the experimental group (*M* = 44 years, ET = 8.71) and the control group (*M* = 36 years, ET = 10.27) were significantly different, and the same was true for the education levels; 45% of the participants in the experimental group had a majority of students with 2–3 years of higher education, while 73% of the students in the control group had a majority of students with 4 years of higher education.

### Procedure

For the first cohort, in order to lead the participant to an IPA understanding of their life experiences, three interviews were scheduled: during their education (T1), 3 months after the end of their education (T2), and 6 months after the education (T3). These three phases were preceded by a preliminary interview to discuss ethical considerations and to obtain free and informed consent.

Designed as a “question pool” used in a flexible and non-exhaustive way, an IPA interview guide structured the three phases, addressing the idiosyncrasy of lived experiences, the influence of the environment (Bernaud, 2018), the components of meaning as a process - directional meaning and meaningful meaning (Bernaud et al., 2020), and life satisfaction (Diener and Tay, 2016). A participant zero helped validate the understanding and consistency of the guide.

For the second cohort, all participants, including those in the control group (Gaudron et al., 2001), individually completed an anonymous self-evaluation questionnaire on the meaning of life and the meaning of education in the pre-test phase 1 week before the beginning of the guidance and in the post-test phase 1 month after exiting the guidance in order to measure the effects. In addition, the participants in the control group received guidance on career choice, but not on the meaning.

### Data Collection

For our first research phase (IPA), a rigorous transcription of the body of interviews was a preliminary step. During the interviews, we also wrote down the emotional expressions. It should be noted that the first transcript was made based on a randomly selected recording (Smith et al., 2009).

The next step consisted of listening to and conscientiously reading the participant’s three interviews in order to identify key words and produce the beginnings of an analysis and understanding. After identifying the theme changes, the body of work was broken down. The items were arranged in a table with three entries: emerging themes, initial transcripts and exploratory comments (descriptive, linguistic, and conceptual). Grouping by themes of meaning present in at least 50% of the sample resulted in the addition of a fourth entry called “clustering.” The procedure was then applied to each of our participants.

For our second research phase (SVSF), two dimensions of meaning were measured by questionnaire: (1) the meaning of life and (2) the meaning of education. The meaning of life dimension consists of the following scales: SWLS (Diener et al., 1985), fulfillment “Flourishing scale” (Diener et al., 2010), MLQ (Steger et al., 2006), and Meaning as a process (Bernaud et al., 2015). The Meaning of Education scale is a variant of the Meaning of Work scale (Bernaud et al., 2015). All items were measured using a seven-point Likert scale, where 1 means “strongly disagree” and 7 means “strongly agree.”

The internal consistency of the scales ranged in the pre-test phase between α = 0.74 and α = 0.89 and in the post-test phase between α = 0.68 and α = 0.85. Only the consistency of the Flourishing scale shows a reliability level in the post-test phase that is slightly lower than α = 0.70 (Table 1).

**TABLE 1:** Pre- and post-test SVSF measurement instrument (*N* = 43).

Dimensions	Scales	Subscales	Pre-test’s α	Post-test’s α
(1) Meaning of life	SWLS		0.84	0.76
	Flourishing scale		0.76	0.68
	MLQ	Search for meaning	0.81	0.84
		Presence of meaning	0.82	0.77
	Inetop meaning scale	Direction	0.89	0.85
		Meaning	0.76	0.75
		Sensation	0.75	0.74
(2) Meaning of education	Education direction	Education direction	0.74	0.70
	Education meaning	Education meaning	0.80	0.70
	Psychological wellbeing	Psychological wellbeing	0.80	0.78
	Joyful learning	Joyful learning	0.78	0.70

## Results

### Analysis of the Participants’ Speech Using the Interpretive Phenomenological Analysis Approach

We will focus here on the elements emerging from the IPA analysis that are most likely to show the links between the meaning of education and psychological wellbeing.

Four main aspects of the meaning of education emerged from the participants’ accounts of their life experiences: (1) An “intrinsic sense of education” dimension, where the learner connects to their needs related to the sensory and perceptual aspects; (2) An “existential meaning of education” dimension, where the learner’s life path and the university’s timeframe are discussed through the prism of the experience of being; (3) An “extrinsic meaning of education” dimension, where the learner develops their relationship with the world and connects them to others; (4) Finally, a “technical meaning of education” dimension, where the learner safely adapts to the environments in which they evolve (Table 2).

**TABLE 2:** Extract of the various emerging themes and grouping by clusters.

Phase 1: Clustering of emerging themes	Phase 2: Thematic clustering
	**I- Existential meaning**
**Meaning of time**	
**Meaning of experience**	
Recollection	
Event experienced as painful Event experienced as traumatic	
**Relationship to timeframe**	
Feeling of wasting time	
Feeling of never having taken the time to know oneself Feeling of not having enough time	
Desire to take time for oneself	
	**Satisfaction with life**
**Search for satisfaction with life**	
Quest for existential success Desire for satisfaction with life	
Self-injunction to satisfaction with life	
	**II- Meaning of education**
	**Search for meaning**
**Reflexivity**	
Reflexivity on education and training	
Reflexivity on the goal to be reached through education	
	**Presence of meaning**
**Level of presence of meaning**	

The “intrinsic meaning of education,” “existential meaning of education” and “extrinsic meaning of education” dimensions and some of their sub-dimensions reveal the unique place of psychological wellbeing in the student’s life. We will illustrate this with verbatims.

The “intrinsic meaning of education” refers to values deeply embedded in and for the self. These values are essential to the individual, independently of their objective utility.

*“Why these studies? because I like to get involved in projects like this that interest me, that I am passionate about.”*

Intrinsic meaning is part of the pleasure-displeasure axis. This dimension encompasses several sub-dimensions, among which we will highlight the joyful learning. Joyful learning is the happiness generated by knowledge in the student, his or her delight in the learning situation or in the idea of learning itself (Nietzsche).

*“I like to learn, I like to study, I’m always up for discovering new things in this training.”*

*“I have a personality that likes to think in the abstract. I have always had a desire to learn, which is why I enrolled in this education.”*

The “existential meaning of education” dimension establishes a link between the act of existing and life satisfaction. The phenomenological approach led each student not only to explore whether their life was satisfying, but to determine through their own history what it meant to have a satisfying life. Throughout their interview, the students detach themselves from a jerky, exhaustive chronological account, which seemed to make up their identity, in order to reach a more elaborated view, allowing them to evaluate with more hindsight the extent to which their life seems satisfying and in relation to what.

The “existential meaning of education” dimension encourages a realization of the authentic self in education, with the student building a new bridge between their existing self and their relationship to the world, allowing them to develop a sense of wellbeing.

*“I think I got into this training unconsciously, and what kept me going is that it makes me exist.”*

This dimension encompasses several sub-dimensions, among which we will highlight here the therapeutic meaning which concerns subjects who have gone through traumatic life situations leading to social exclusion or emotional difficulties (Gergen, 2015). The therapeutic meaning encompasses themes such as: flourishing (Guimard et al., 2015), distress, recognition, self-confidence, self-esteem, and identity.

*“I need to take this course to have more confidence in myself et al. so in relation to the way others and society look at me.”*

*“I wanted to study. to be somebody. To be someone is to know things, to be respected, not to be a loser.”*

*“Thanks to the training I have reconstructed myself, I have discovered myself with strengths that I never imagined. I think that this training course is somewhere to repair what was left behind, what was neglected 12 years ago.”*

The “extrinsic meaning of education” dimension also includes various sub-dimensions, among which we will point out the eudemonic meaning, or the search for achieving prescribed goals that contribute to the wellbeing of the family and the community (Seligman, 2002). Here the student aspires to build their academic and professional life orientation from the perspective of maintaining the wellbeing of their family unit. In general, the IPA reveals that family demands may have been destabilizing, even distressing, for these students, who have given up, at least temporarily, on affirming themselves as existing beings, in order to give themselves to those around them. This is their priority, which will perhaps later be reversed in favor of a subsequent consideration of the self, of one’s own psychological wellbeing.

*“With this training I want to do better than my parents did for them. To say that they sacrificed for something. I feel indebted to my parents.”*

*“This training proposed by my mother has allowed me to see many things and to have another way of thinking, to be well.”*

### Analysis of SVSF Device Effects by Hegde’s *g*

Applying Hedge’s *g*, appropriate for our sample sizes (Watson et al., 2016), reveals an overall average effect of *g* = + 0.69 for the experimental sample (*N* = 29) (Table 3). Thus, all of Hedge’s g indicators, measuring the strength of a change for SVSF participants, confirm the presence of effects. SVSF affects meaning of life (*g* = + 0.69), meaning of education (*g* = + 0.73), life satisfaction (*g* = + 0.78), sense of fulfillment (*g* = + 0.71), search for meaning (*g* = + 0.86), presence of meaning (*g* = + 0.81), direction in life (*g* = + 0.72), the meaning given to their education (*g* = + 1.05), the direction given to their education (*g* = + 0.72), and the feeling of psychological wellbeing in education (*g* = + 0.66). Questioning the relationship between the meaning given to one’s life and one’s education is therefore relevant. They also confirm that questioning meaning leads the individual to overcome obstacles encountered in his or her personal life and/or education.

**TABLE 3:** Measurement of Hedge *g* effects on the experimental group.

Experimental group	Scales	Subscale	Hedge g	Interpretation of effect
(1) Meaning of life	SWLS		0.78	Medium
	Flourishing scale		0.71	Medium
	MLQ	Search for meaning	**0.86**	**Large**
		Presence of meaning	**0.81**	**Large**
	Inetop meaning scale	Direction	0.72	Medium
		Meaning	0.38	Small
		Sensation	0.44	Small
(2) Meaning of education	Education direction		0.72	Medium
	Education meaning		**1.05**	**Large**
	Psychological wellbeing		0.66	Medium
	Joyful learning		0.47	Small

*Experimental sample (N = 29); g average = + 0.69; 0.01 = very small; 0.20 = small; 0.5 = medium; 0.8 = large; 1.20 = very large; and 2.00 = huge.*

Concerning the control group (*N* = 11), the results obtained indicate a global average effect of *g* = + 0.36, which results in a small size effect (Table 4). More specifically, the overall results of the control group show a significantly lower level of meaning of life and a significantly lower level of meaning of education than the experimental group. Thus, the SVSF guidance would have contributed to an important and significant improvement in the meaning attributed by the participants to their life and their education. Similarly, the overall results of the control group show a significantly lower level of wellbeing and life satisfaction than the experimental group. SVSF would therefore also have contributed to a significant improvement in the participants’ sense of wellbeing and life satisfaction.

**TABLE 4:** Measurement of Hedge *g* effects on the control group.

Experimental group	Scales	Subscales	Hedge *g*	Interpretation of effect
(1) Meaning of life	SWLS		0.31	Small
	Flourishing scale		0.40	Small
	MLQ	Search for meaning	0.24	Small
		Presence of meaning	0.65	Medium
	Inetop meaning scale	Direction	0.26	Small
		Meaning	0.04	Very small
		Sensation	0.29	Small
(2) Meaning of education	Education direction		0.24	Small
	Education meaning		0.06	Very small
	Psychological wellbeing		0.52	Medium
	Joyful learning		0.95	**Large**

*Experimental sample (N = 11); g average = + 0.36; 0.01 = very small; 0.20 = small; 0.5 = medium; 0.8 = large; 1.20 = very large; and 2.00 = huge.*

## Discussion

Our literature review has shown that students can be faced with stress leading to a feeling of malaise and a risk of losing meaning during their university education. The societal context in which they evolve changes their relationship to learning and increases the risk of the appearance of pathogenic phenomena such as workaholism, hikikomori, and burnout.

A student’s wellbeing is not a luxury; it validates the meaning attributed to their plans, it reveals their psychological health, and is a predictor of their commitment. How can we foster or consolidate this wellbeing? Through reflective work on the meaning that the student attributes to their education.

Implementing an educational plan is not simply a matter of receiving education; it is often the expression of a desire to belong to the world, the expression of an existential desire. Life history and education are intimately linked. Education appears as the realization of a work subject to unconscious inner movements and to a desire to be part of the world. Reflective work on their major life experiences allows the student to become aware of why they agreed to studying and the meaning they attribute to it. The mechanisms of choice are made manifest and potentially transformed. This promotes the stability of their educational commitment.

At the end of this research, we can consider our hypothesis H1 as valid: “Seeking the meaning of an education through a reflective guidance would increase the student’s sense of psychological wellbeing by promoting more authentic choices.” The quantitative data from SVSF confirms this, showing significantly strong effects for the Life Satisfaction, Flourishing and Psychological wellbeing.

Moreover, we can consider our hypothesis H2 as valid: “A reflective approach to one’s life experiences would enable the student to clarify their choices and attribute a high level of meaning to their education.” Our two research periods were able to confirm this, through the IPA verbatims and the SVSF quantitative data. By giving meaning to their education, objectified by distancing themselves from their emotions and beliefs, the IPA offers students the freedom to choose. As for SVSF, it shows significantly strong effects for Meaning of Life and for Meaning of Training, including Education direction.

Both IPA and SVSF engage the individual in a reflexive process, a process of co-evaluation of his perception of his major life experiences (Jorro, 2005) with the aim of encouraging awareness of his essence (Husserl, 2004). During these two periods, the students were led to shed light on certain shadowy areas of their experience. They were in the position of co-researchers, people who are beings in the world (Heidegger, 1986) and who, through a reflexive posture, are able to understand their own perceptions of meaning and meaninglessness in their lives and learning experience (Merleau-Ponty, 1945).

By “capturing” the major life experiences that the student has struggled to interpret unsuccessfully on their own, agreeing to reflective work (IPA or SVSF) offers them a better education-life balance and greater emotional stability. Indeed, in this transformative process, the sources of the student’s malaise.

are discovered, and the attribution of new meanings, leading to new choices and liberating behaviors, resulting in a lightening of the mental load.

IPA, an approach to the study of complex psychological phenomena, has also proven to be a remarkable support to meaning. However, because it is rooted in a community of peers, the reflective work agreed upon in SVSF enables the individual to progress with a certain delicacy from a progressively conscious self to a secure and gratifying person in the world.

### Practical Implications

Our research is not part of a therapeutic or prescriptive approach. It aims to lead the student to adopt an active stance, resulting in a descriptive, analytical and interpretative mental process of their lived experiences. University guidance is traditionally based on the use of psychometric assessments (e.g., interest questionnaires). The counselor then positions himself as a mediator between the tool and the student, without questioning the meaning for the student of his education. This limits the strength of a vocational choice. This is why we wish to demonstrate the potential of a guidance system to clarify the meaning of education.

Indeed, it is important in our opinion to enrich the reflection of the universities and counseling and guidance services for students. The university should support the students in the development of this meaning through concern for their psychological needs, considering also that their success depends largely on it. The challenge of providing support for meaning is to prevent any risk of loss of meaning and a feeling of malaise that can lead to a feeling of existential emptiness. Understanding one’s relationship to the world, to others, to oneself, to one’s education, inevitably leads the student to live a more authentic relationship with the latter and to develop a feeling of wellbeing.

### Limitations and Futures Perspectives

It would be interesting to be able to study the potential differences in perception between learners by academic year. A breakdown by age group would also be feasible. However, such an approach would require a significant increase in our two cohorts (IPA and SVSF).

Using larger samples would also allow a comparison of the effects of a reflective approach on initial and continuing education students.

Another limitation is that this same research was conducted over one academic year. It would be interesting to develop a new timeframe by conducting the IPA interviews and the SVSF support system over a full academic cycle to test for effects and stability over a longer period (Forgues and Vandangeon-Derumez, 1999).

Finally, before starting this research, we did not specifically intend to use the IPA approach as a support device, but its potential in this respect could lead us to think of a detailed structuring between these two modes of developing reflexivity.

## Data Availability Statement

The raw data supporting the conclusions of this article will be made available by the authors, without undue reservation.

## Ethics Statement

Ethical review and approval was not required for the study on human participants in accordance with the local legislation and institutional requirements. The patients/participants provided their written informed consent to participate in this study.

## Author Contributions

All authors conducted the literature search, analyzed and processed the data, and wrote and reviewed the manuscript.

## Conflict of Interest

The authors declare that the research was conducted in the absence of any commercial or financial relationships that could be construed as a potential conflict of interest.

## Publisher’s Note

All claims expressed in this article are solely those of the authors and do not necessarily represent those of their affiliated organizations, or those of the publisher, the editors and the reviewers. Any product that may be evaluated in this article, or claim that may be made by its manufacturer, is not guaranteed or endorsed by the publisher.
